# Supplementary material for: Bamboo Shoots Modulate Gut Microbiota, Eliminate Obesity in High-Fat-Diet-Fed Mice and Improve Lipid Metabolism
Source: Foods. 2023 Mar 24;12(7):1380. doi: 10.3390/foods12071380 (PMC10093345; doi:10.3390/foods12071380)
Supplement: Supplementary file 1 [file foods-12-01380-s001.zip › foods-2242752-supplementary.pdf]

Supplemental file

Bamboo Shoots Modulate Gut Microbiota, Eliminate Obesity in High-Fat-Diet-Fed Mice and Improve Lipid Metabolism

Xiaolu Zhou, SolJu Pak, Daotong Li, Li Dong, Fang Chen, Xiaosong Hu and Lingjun Ma *

National Engineering Research Center for Fruit and Vegetable Processing, Key Laboratory of Fruit and
Vegetables Processing Ministry of Agriculture, College of Food Science and Nutritional Engineering, China Agricultural University, Beijing 100083, China; zxl782072778@163.com (X.Z.); ogj1991928@163.com (S.P.); lidaotong@bjmu.edu.cn (D.L.); li_dong127@163.com (L.D.); chenfangch@sina.com (F.C.);
huxiaos@263.net (X.H.)

* Correspondence: lingjun.ma@cau.edu.cn; Tel.: +86-13693082607

This file includes:

Figure S1. Effects of BS on (A) energy intake, (B) adipose cell size and (C) Liver histological score.

Figure S2. Effects of BS on the gut microbiota of mice at the phylum level: (A) Firmicutes, (B) Bacteroidota, (C) Proteobacteria, (D) Desulfobacterota and (E) Verrucomicrobia.

Figure S3. Cladogram generated from LEfSe analysis showing the relationship among taxa: (A) HFD vs. NCD (B) HFD-BS vs. HFD.

Figure S4. Linear discriminant analysis (LDA) scores derived from LEfSe analysis, showing the biomarker taxa LDA score of >3. (A) HFD vs. NCD (B) HFD-BS vs. HFD.

Table S1. Composition of bamboo shoot freeze-dried powder (g/100g).

Table S2. Reagents for experiment.

Table S3. Composition of experimental diets.

Table S4. Liver steatosis evaluation form.

Method S1. Gut microbiota analysis.

Method S2. Quantification of fecal short-chain fatty acids (SCFAs).

Method S3. Determination of metabolites in faces.

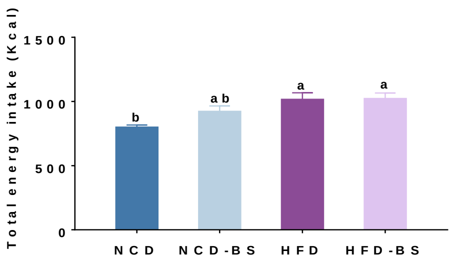

(A)

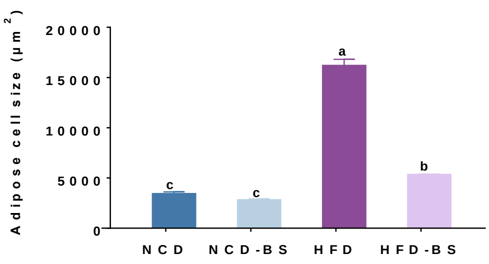

(B)

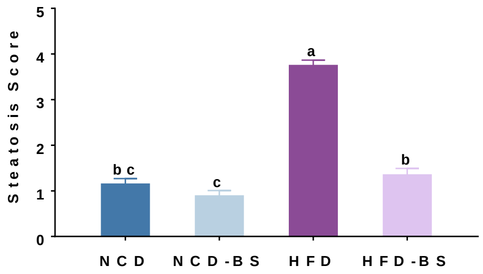

(C)

Figure S1. **Effects of BS on (A) energy intake, (B) adipose cell size and (C) Liver histological score.** Data presented as mean ± SEM, n = 8 per group. Means denoted by a different letter (a, b, c) indicate significant differences between groups ($p < 0.05$). NCD, normal control diet; NCD-BS, normal control diet supplemented with freeze-dried powder of bamboo shoots; HFD, high-fat diet; HFD-BS, high-fat diet supplemented with freeze-dried powder of bamboo shoots.

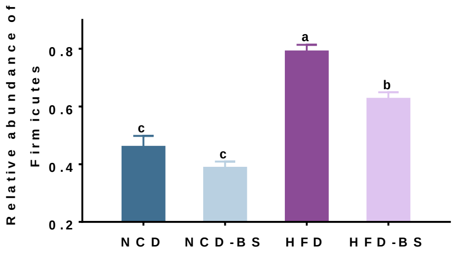

(A)

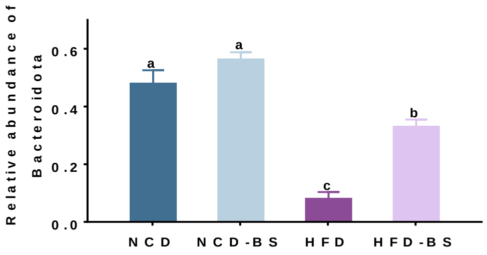

(B)

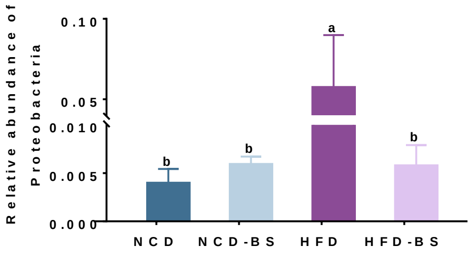

(C)

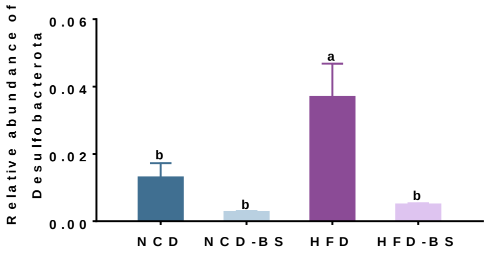

(D)

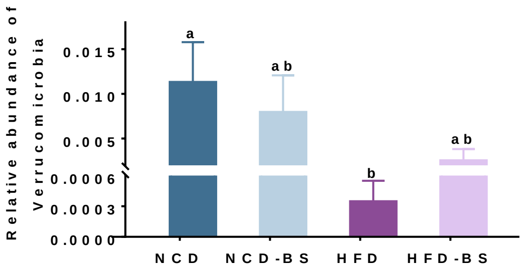

(E)

Figure S2. **Effects of BS on the gut microbiota of mice at the phylum level: (A) Firmicutes, (B) Bacteroidota, (C) Proteobacteria, (D) Desulfobacterota and (E) Verrucomicrobia.** Data presented as mean ± SEM, n = 8 per group. Means denoted by a different letter (a, b, c) indicate significant differences between groups ($p < 0.05$). NCD, normal control diet; NCD-BS, normal control diet supplemented with freeze-dried powder of bamboo shoots; HFD, high-fat diet; HFD-BS, high-fat diet supplemented with freeze-dried powder of bamboo shoots.

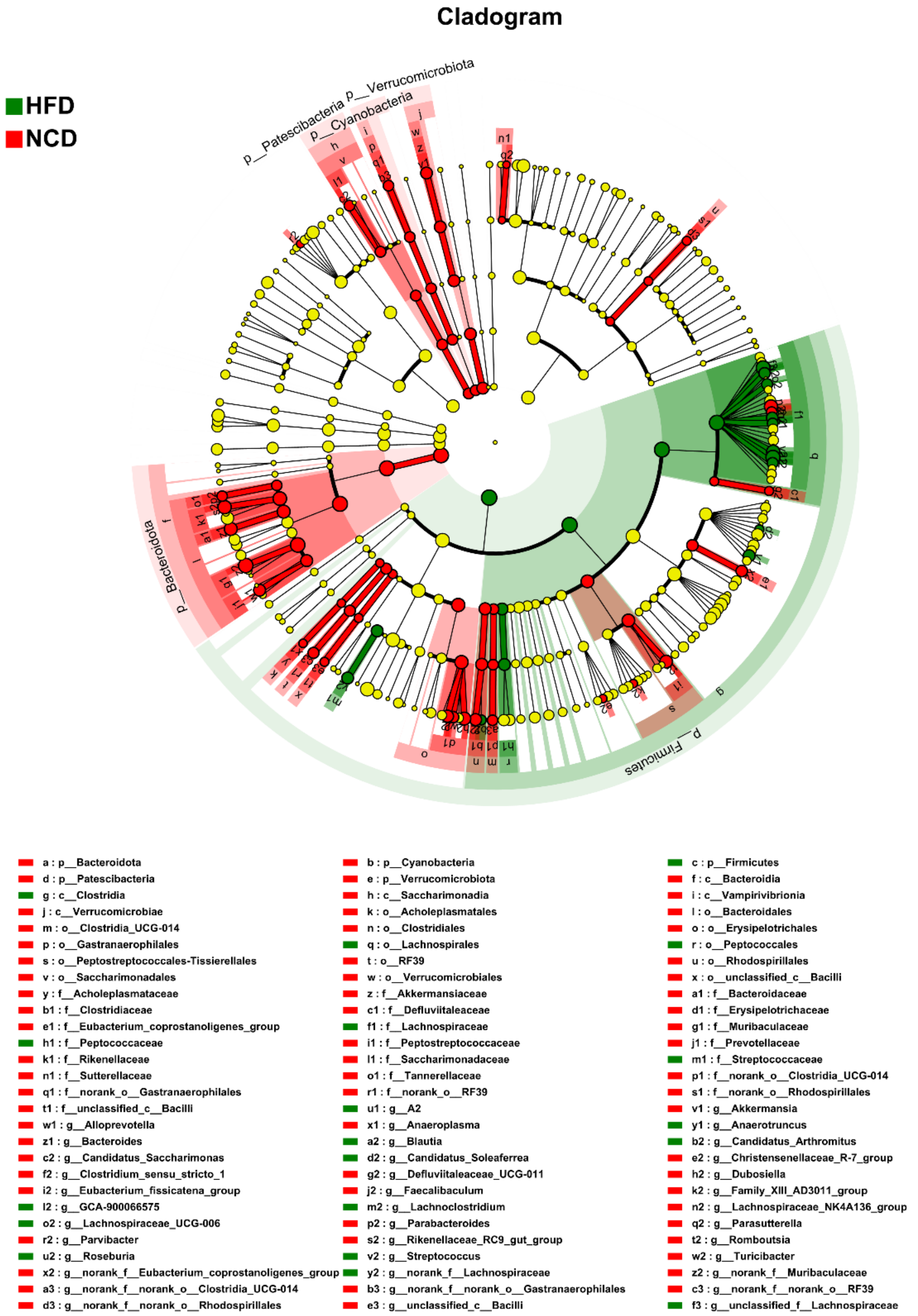
Cladogram
HFD
NCD
p__Patescibacteria
p__Verrucomicrobiota
p__Cyanobacteria
p__Bacteroidota
p__Firmicutes
a : p__Bacteroidota
b : p__Cyanobacteria
c : p__Firmicutes
d : p__Patescibacteria
e : p__Verrucomicrobiota
f : c__Bacteroidia
g : c__Clostridia
h : c__Saccharimonadia
i : c__Vampirivibrionia
j : c__Verrucomicrobiae
k : o__Acholeplasmatales
l : o__Bacteroidales
m : o__Clostridia_UCG-014
n : o__Clostridiales
o : o__Erysipelotrichales
p : o__Gastranaerophilales
q : o__Lachnospirales
r : o__Peptococcales
s : o__Peptostreptococcales-Tissierellales
t : o__RF39
u : o__Rhodospirillales
v : o__Saccharimonadales
w : o__Verrucomicrobiales
x : o__unclassified_c__Bacilli
y : f__Acholeplasmataceae
z : f__Akkermansiaceae
a1 : f__Bacteroidaceae
b1 : f__Clostridiaceae
c1 : f__Defluviitaleaceae
d1 : f__Erysipelotrichaceae
e1 : f__Eubacterium_coprostanoligenes_group
f1 : f__Lachnospiraceae
g1 : f__Muribaculaceae
h1 : f__Peptococcaceae
i1 : f__Peptostreptococcaceae
j1 : f__Prevotellaceae
k1 : f__Rikenellaceae
l1 : f__Saccharimonadaceae
m1 : f__Streptococcaceae
n1 : f__Sutterellaceae
o1 : f__Tannerellaceae
p1 : f__norank_o__Clostridia_UCG-014
q1 : f__norank_o__Gastranaerophilales
r1 : f__norank_o__RF39
s1 : f__norank_o__Rhodospirillales
t1 : f__unclassified_c__Bacilli
u1 : g__A2
v1 : g__Akkermansia
w1 : g__Alloprevotella
x1 : g__Anaeroplasma
y1 : g__Anaerotruncus
z1 : g__Bacteroides
a2 : g__Blautia
b2 : g__Candidatus_Arthromitus
c2 : g__Candidatus_Saccharimonas
d2 : g__Candidatus_Soleaferrea
e2 : g__Christensenellaceae_R-7_group
f2 : g__Clostridium_sensu_stricto_1
g2 : g__Defluviitaleaceae_UCG-011
h2 : g__Dubosiella
i2 : g__Eubacterium_fissicatena_group
j2 : g__Faecalibaculum
k2 : g__Family_XIII_AD3011_group
l2 : g__GCA-900066575
m2 : g__Lachnoclostridium
n2 : g__Lachnospiraceae_NK4A136_group
o2 : g__Lachnospiraceae_UCG-006
p2 : g__Parabacteroides
q2 : g__Parasutterella
r2 : g__Parvibacter
s2 : g__Rikenellaceae_RC9_gut_group
t2 : g__Romboutsia
u2 : g__Roseburia
v2 : g__Streptococcus
w2 : g__Turicibacter
x2 : g__norank_f__Eubacterium_coprostanoligenes_group
y2 : g__norank_f__Lachnospiraceae
z2 : g__norank_f__Muribaculaceae
a3 : g__norank_f__norank_o__Clostridia_UCG-014
b3 : g__norank_f__norank_o__Gastranaerophilales
c3 : g__norank_f__norank_o__RF39
d3 : g__norank_f__norank_o__Rhodospirillales
e3 : g__unclassified_c__Bacilli
f3 : g__unclassified_f__Lachnospiraceae

(**A**)

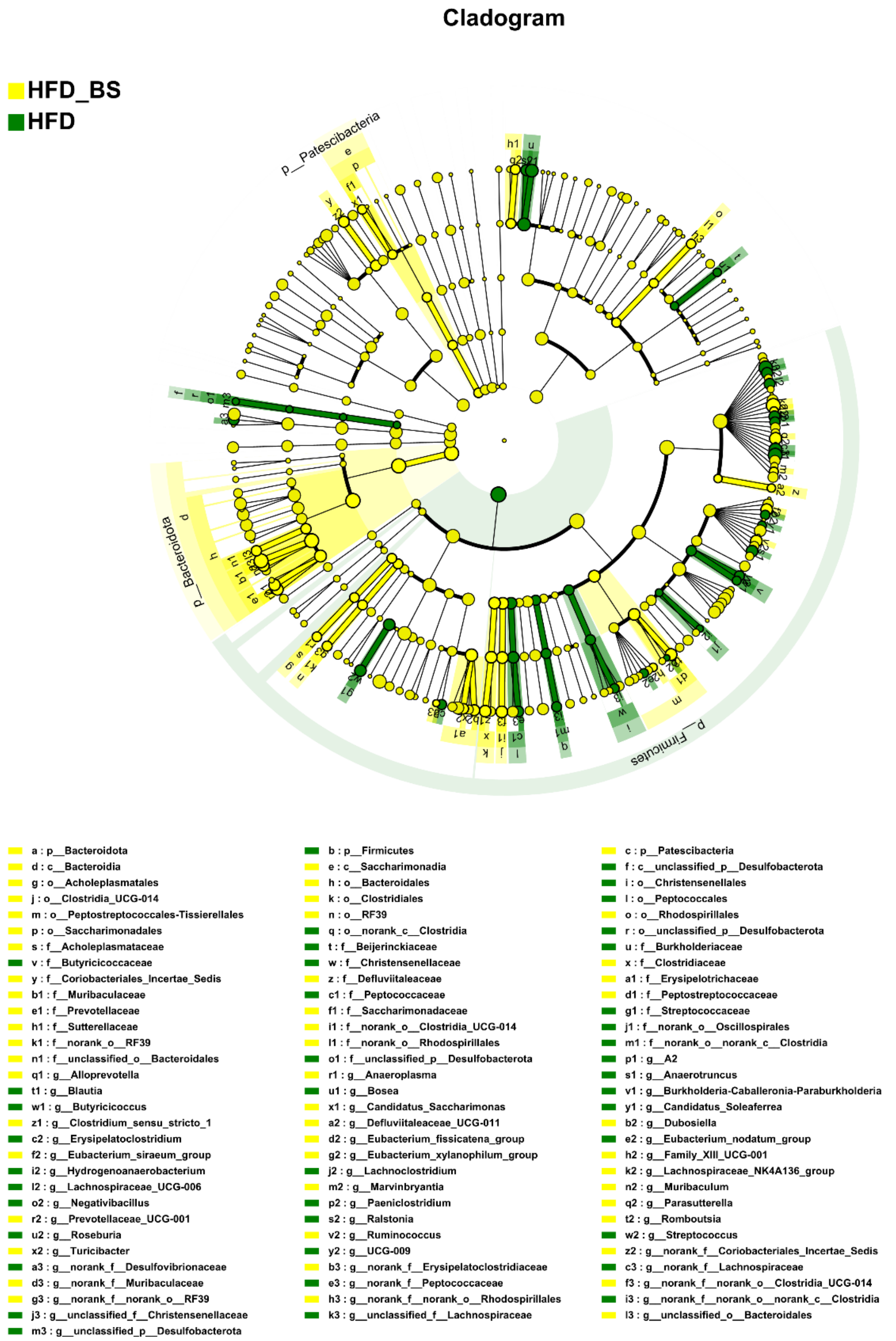

(B)

Figure S3. Cladogram generated from LEfSe analysis showing the relationship among taxa: (A) HFD vs. NCD (B) HFD-BS vs. HFD. LEfSe were assessed with the non-parametric factorial Kruskal–Wallis (KW) sum-rank test and show the abundances of the gut microbiota at the phylum to genus level. The differently colored nodes indicate microbial taxa that are

significantly enriched in the corresponding groups and have a significant effect on the differences between groups; the light-yellow nodes indicate microbial taxa that are not significantly different in any of the different groups or have no significant effect on the differences between groups. n =8 per group. NCD, normal control diet; NCD-BS, normal control diet supplemented with freeze-dried powder of bamboo shoots; HFD, high-fat diet; HFD-BS, high-fat diet supplemented with freeze-dried powder of bamboo shoots.

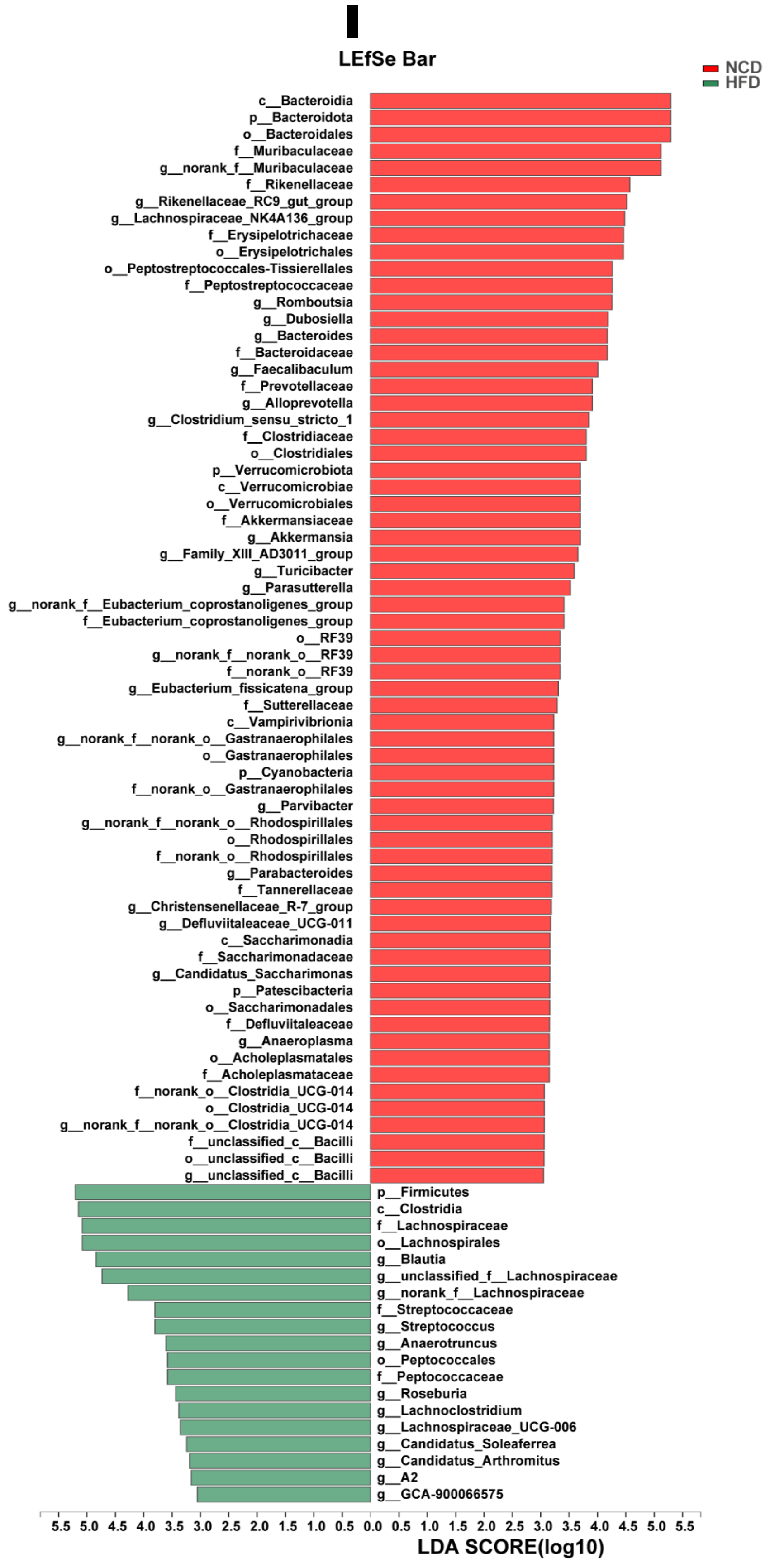
LEfSe Bar
NCD
HFD
c__Bacteroidia
p__Bacteroidota
o__Bacteroidales
f__Muribaculaceae
g__norank_f__Muribaculaceae
f__Rikenellaceae
g__Rikenellaceae_RC9_gut_group
g__Lachnospiraceae_NK4A136_group
f__Erysipelotrichaceae
o__Erysipelotrichales
o__Peptostreptococcales-Tissierellales
f__Peptostreptococcaceae
g__Romboutsia
g__Dubosiella
g__Bacteroides
f__Bacteroidaceae
g__Faecalibaculum
f__Prevotellaceae
g__Alloprevotella
g__Clostridium_sensu_stricto_1
f__Clostridiaceae
o__Clostridiales
p__Verrucomicrobiota
c__Verrucomicrobiae
o__Verrucomicrobiales
f__Akkermansiaceae
g__Akkermansia
g__Family_XIII_AD3011_group
g__Turicibacter
g__Parasutterella
g__norank_f__Eubacterium_coprostanoligenes_group
f__Eubacterium_coprostanoligenes_group
o__RF39
g__norank_f__norank_o__RF39
f__norank_o__RF39
g__Eubacterium_fissicatena_group
f__Sutterellaceae
c__Vampirivibrionia
g__norank_f__norank_o__Gastranaerophilales
o__Gastranaerophilales
p__Cyanobacteria
f__norank_o__Gastranaerophilales
g__Parvibacter
g__norank_f__norank_o__Rhodospirillales
o__Rhodospirillales
f__norank_o__Rhodospirillales
g__Parabacteroides
f__Tannerellaceae
g__Christensenellaceae_R-7_group
g__Defluviitaleaceae_UCG-011
c__Saccharimonadia
f__Saccharimonadaceae
g__Candidatus_Saccharimonas
p__Patescibacteria
o__Saccharimonadales
f__Defluviitaleaceae
g__Anaeroplasma
o__Acholeplasmatales
f__Acholeplasmataceae
f__norank_o__Clostridia_UCG-014
o__Clostridia_UCG-014
g__norank_f__norank_o__Clostridia_UCG-014
f__unclassified_c__Bacilli
o__unclassified_c__Bacilli
g__unclassified_c__Bacilli
p__Firmicutes
c__Clostridia
f__Lachnospiraceae
o__Lachnospirales
g__Blautia
g__unclassified_f__Lachnospiraceae
g__norank_f__Lachnospiraceae
f__Streptococcaceae
g__Streptococcus
g__Anaerotruncus
o__Peptococcales
f__Peptococcaceae
g__Roseburia
g__Lachnoclostridium
g__Lachnospiraceae_UCG-006
g__Candidatus_Soleaferrea
g__Candidatus_Arthromitus
g__A2
g__GCA-900066575
5.5 5.0 4.5 4.0 3.5 3.0 2.5 2.0 1.5 1.0 0.5 0.0 0.5 1.0 1.5 2.0 2.5 3.0 3.5 4.0 4.5 5.0 5.5
LDA SCORE(log10)

(**A**)

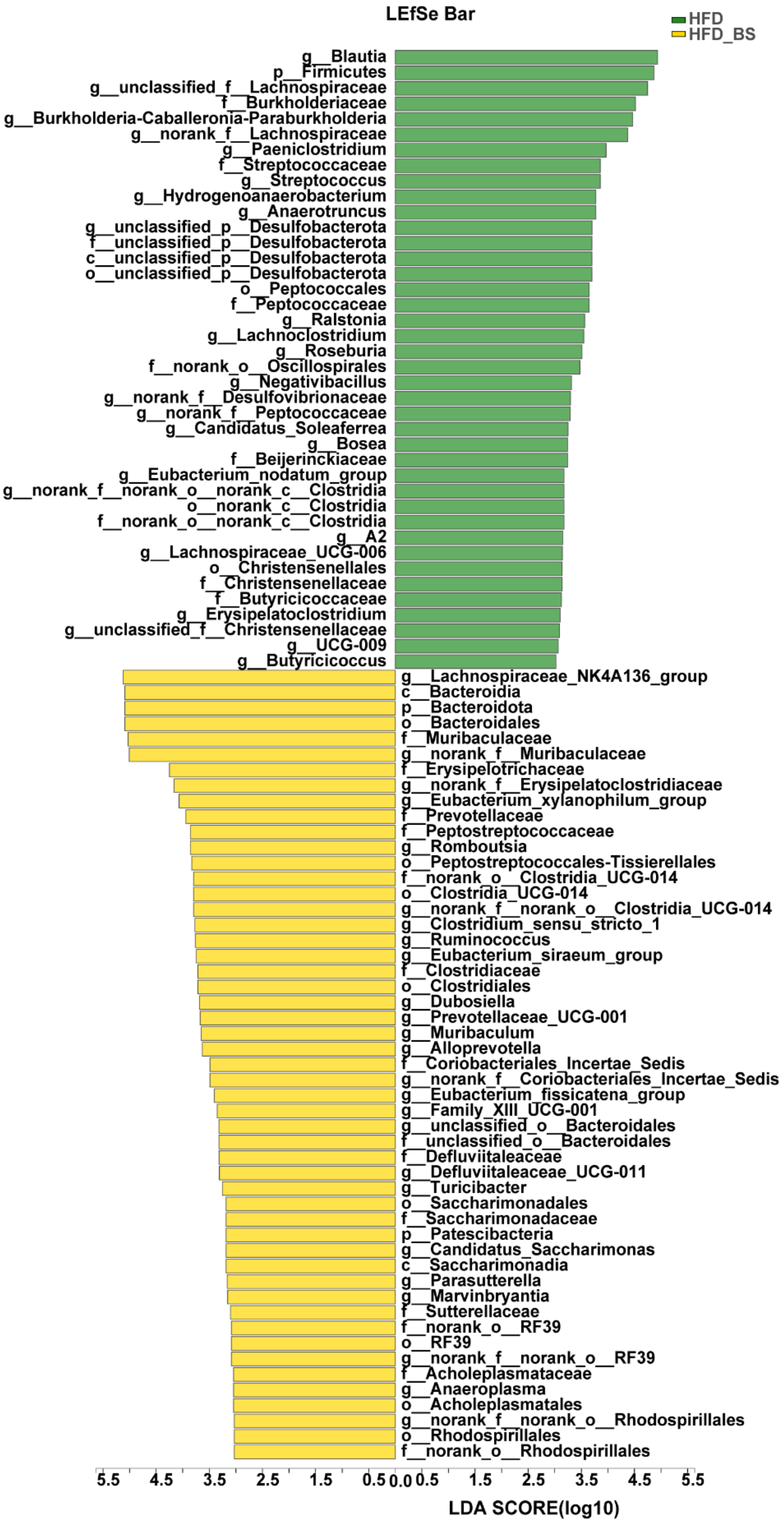

(**B**)

Figure S4. Linear discriminant analysis (LDA) scores derived from LEfSe analysis, showing a biomarker taxa LDA score of >3. (A) HFD vs. NCD (B) HFD-BS vs. HFD. LDA discriminant

bar chart by LDA scores obtained via LDA analysis (linear regression analysis); the higher the LDA score, the greater the effect of species abundance on the differential effect. It was possible to count the microbial taxa that had a significant effect in multiple groups. n =8 per group. NCD, normal control diet; NCD-BS, normal control diet supplemented with freeze-dried powder of bamboo shoots; HFD, high-fat diet; HFD-BS, high-fat diet supplemented with freeze-dried powder of bamboo shoots.

Table S1. Composition of bamboo shoot freeze-dried powder (g/100g).

Nutrient content	Dry weight
Total dietary fiber	26. 60±0. 40
Insoluble dietary fiber	25. 70±1. 17
Soluble dietary fiber	0. 90±1. 56
Protein	41. 80±0. 99
Fat	2. 43±0. 03
Carbohydrate	12. 17±0. 60
Moisture	2. 90±0. 06
Ash	14. 10±0. 04

Data are expressed as mean ± standard deviation (n = 3).

Table S2. Reagents for experiment.

Reagent	Manufacturer	Purity level
Acetic acid	Sigma-aldrich Company, USA	chromatographic grade
Propionic acid	Sigma-aldrich Company, USA	chromatographic grade
Butyric acid	Sigma-aldrich Company, USA	chromatographic grade
Isobutyric acid	Sigma-aldrich Company, USA	chromatographic grade
Pentanoic acid	Sigma-aldrich Company, USA	chromatographic grade
Isovaleric acid	Sigma-aldrich Company, USA	chromatographic grade
Diethyl ether	Sinopharm Chemical Reagent Co	analytical grade
Anhydrous ethanol	Sinopharm Chemical Reagent Co	analytical grade
Methanol	Sinopharm Chemical Reagent Co	analytical grade

Table S3. Composition of experimental diets[a].

Ingredient (g/kg)	NCD[b]	NCD-BS	HFD	HFD-BS
Lyophilized powder of bamboo shoots	0	150.00	0	150.00
Casein, 80 Mesh	189. 56	114. 02	258. 45	182. 90
L-Cystine	2. 84	2. 84	3. 88	3. 88
Corn Starch	479. 79	457. 11	0. 00	0. 00
Maltodextrin 10	118.48	118.48	161. 53	139. 55
Sucrose	73. 74	73. 74	88. 91	88. 91
Cellulose	47. 39	0.00	64. 61	16. 54
Soybean Oil	23. 70	19. 31	32. 31	27. 91
Lard	18. 96	18. 96	316. 60	316. 60
Mineral Mix, S10026	9. 48	9. 48	12. 92	12. 92
DiCalcium Phosphate	12. 32	12. 32	16. 80	16. 80
Calcium Carbonate	5. 21	5. 21	7. 11	7. 11
Potassium Citrate, 1 H20	15. 64	15. 64	21. 32	21. 32
Vitamin Mix, V10001C	0. 95	0. 95	12. 92	12. 92
Choline Bitartrate	1. 90	1. 90	2.58	2.58
FD&C Blue Dye	0. 01	0. 01	0. 00	0. 00
FD&C Yellow Dye	0. 04	0. 04	0. 00	0. 00
FD&C Blue Dye #1	0. 00	0. 00	0. 005	0. 005
% Energy and source				
Protein	20	20	20	20
Carbohydrate	70	70	20	20
Fat	10	10	60	60

[a] Diet formulae of NCD and HFD. The ingredients were obtained from Shuyishuer Biotech Co., Ltd, Changzhou, China.

[b] NCD, normal control diet; NCD-BS, normal control diet supplemented with Qiong bamboo shoot freeze-dried powder; HFD, high-fat diet; HFD-BS, high-fat diet supplemented with Qiong bamboo shoot freeze-dried powder.

Table S4. Liver steatosis evaluation form.

Degree of Liver Steatosis	Score	Evaluation criteria
Normal	0.0-1.0	The liver peritoneum is intact, the whole liver is stained pink, and the boundaries between hepatocyte cords are clear; the hepatocytes are slightly swollen, the cytoplasmic boundaries are obvious, the intracytoplasmic granularity is not obvious, and small vacuoles (formed by the dissolution of lipid droplets) occasionally appear in the cytoplasm of individual cells.
Mild	1.0-2.0	The liver is intact, the whole liver is stained pink, the intercellular boundary of hepatocytes is clear; the hepatocytes are swollen to some extent, the cytoplasmic boundary is still obvious, the intracytoplasmic granularity is obvious, some cells have small vacuoles in the cytoplasm, and the number of vacuoles is obviously increased compared with a normal liver.
Moderate	2.0-3.0	The liver is intact, the whole liver is stained light pink, the intercellular boundary of hepatocytes is still clear; the hepatocytes are obviously swollen, the cytoplasmic boundary is no longer obvious, the intracytoplasmic granularity is obvious, the sinusoidal gap is narrowed, many small vacuoles appear in the cytoplasm of most cells, and some of the small vacuoles have fused to form larger vacuoles.
Severe	3.0-4.0	The liver is intact, the whole liver is stained with a very light pink color, the boundary between hepatocyte cords is not very clear; the hepatocytes are highly swollen, the cytoplasmic boundary is no longer obvious, the sinusoidal gap is almost gone, there are large vacuoles (formed by the fusion of many vacuoles after the dissolution of small lipid droplets) in the cytoplasm of most of the cells.

Method S1. Gut Microbiota Analysis.

Total microbial genomic DNA was extracted from fecal samples using the E.Z.N.A.® soil DNA Kit (Omega Bio-tek, Norcross, GA, U.S.) according to manufacturer's instructions. The quality and concentration of DNA were determined via 1.0% agarose gel electrophoresis and a NanoDrop® ND-2000 spectrophotometer (Thermo Scientific Inc., USA) and kept at -80 °C prior to further use. The hypervariable region V3-V4 of the bacterial 16S rRNA gene were amplified with primer pairs 338F (5'-ACTCCTACGGGAGGCAGCAG-3') and 806R (5'-GGACTACHVGGGTWTCTAAT-3') by an ABI GeneAmp® 9700 PCR thermocycler (ABI, CA, USA). The PCR reaction mixture included 4 μL of 5 × Fast Pfu buffer, 2 μL of 2.5 mM dNTPs, 0.8 μL of each primer (5 μM), 0.4 μL of Fast Pfu polymerase, 10 ng of template DNA and ddH2O with a final volume of 20 μL. PCR amplification cycling conditions were as follows: initial denaturation at 95 °C for 3 min, followed by 27 cycles of denaturing at 95 °C for 30 s, annealing at 55 °C for 30 s and extension at 72 °C for 45 s, and single extension at 72 °C for 10 min and ending at 4 °C. All samples were amplified in triplicate. The PCR product was extracted from 2% agarose gel and purified using the AxyPrep DNA Gel Extraction Kit (Axygen Biosciences, Union City, CA, USA) according to the manufacturer's instructions and quantified using Quantus™ Fluorometer (Promega, USA).

Purified amplicons were pooled in equimolar amounts and pair-end-sequenced on an Illumina MiSeq PE300 platform/NovaSeq PE250 platform (Illumina, San Diego, USA) according to the standard protocols of Majorbio Bio-Pharm Technology Co. Ltd. (Shanghai, China).

Raw FASTQ files were de-multiplexed using an in-house Perl script, and then quality-filtered using fastp version 0.19.6 and merged using FLASH version 1.2.7 with the following criteria: (i) the 300 bp reads were truncated at any site receiving an average quality score of < 20 over a 50 bp sliding window, and truncated reads shorter than 50 bp and reads containing ambiguous characters were discarded; (ii) only overlapping sequences longer than 10 bp were assembled according to their overlapped sequence. The maximum mismatch ratio of overlap region was 0.2. Reads that could not be assembled were discarded; (iii) samples were distinguished according to barcode and primers, and the sequence direction was adjusted via exact barcode matching, with two nucleotide mismatches found in primer matching. Then, the optimized sequences were clustered into operational taxonomic units (OTUs) using UPARSE 7.1 with 97% sequence similarity level. The most abundant sequence for each OTU was selected as a representative sequence. The OTU table was manually filtered, i.e., chloroplast sequences in all samples were removed. To minimize the effects of sequencing depth on alpha and beta diversity, the number of 16S rRNA gene sequences from each sample was rarefied to 20,000, which still yielded an average Good's coverage of 99.09%, respectively. The taxonomy of each OTU representative sequence was analyzed via RDP Classifier version 2.2 against the 16S rRNA gene database (e.g., Silva v138) using a confidence threshold of 0.7.

Bioinformatic analysis of the gut microbiota was carried out using the Majorbio Cloud platform (https://cloud.majorbio.com). The alpha diversity analysis was performed using Mothur (version V. 1. 30. 2). Both nonmetric multidimensional scaling (NMDS) and principal coordinate analysis (PCoA) were applied to quantify the compositional differences between the microbial communities based on Bray–Curtis dissimilarity using Vegan v2.5-3 package. ANOSIM analysis was used to test for significant differences in clusters among the groups. The relative abundances of different bacterial communities were assessed with the Wilcoxon rank-sum test at a confident level of 95%, which was corrected using a false discovery rate (FDR). Linear discriminant analysis (LDA) effect size (LEfSe) (http://huttenhower.sph.harvard.edu/LEfSe) was performed to identify the significantly abundant taxa (phylum to genus) of bacteria among the different groups (LDA score > 3, p <

0.05, FDR corrected). LEfSe were assessed with the non-parametric factorial Kruskal–Wallis (KW) sum-rank test.

Method S2. Quantification of fecal short-chain fatty acids (SCFAs).

SCFAs were first extracted by mixing 100 mg of fresh mouse feces with 800 μL of distilled water, 200 μL of 50% concentrated sulfuric acid and 1 mL of ether. The mixture was shaken for 5 min, vortexed and centrifuged at 12,000 x g for 10 min, followed by dehydration with anhydrous $CaCl_2$ and aspiration of the supernatant over a membrane (0.22 μm, nylon membrane). The analysis was carried out via Shimadzu gas chromatography using an SH-Stabilwax-DA capillary column (30 m × 0.32 mm × 0.25 μm) with a solvent delay time of 2 min. The initial temperature was held at 90 °C for 2 min, followed by an increase to 220 °C at 15 °C/min for 5 min. The detector temperature was set at 175 °C and the carrier gas was He (1.0 mL/min). Data were acquired using SIM mode and Shimadzu workstation software.

Acetic acid, propionic acid, butyric acid, isobutyric acid, n-valeric acid and isovaleric acid standards were diluted to 10-1,000 ppm. A total of 50 μL of the standard mixture and 50 μL of the internal reference (400 μg/mL 2-ethylbutyric acid) were mixed for gas-phase analysis to produce a standard curve, and the samples were calculated from the standard curve in mg/g.

Method S3. Determination of metabolites in faces.

A total of 50 mg of solid sample was accurately weighed, and the metabolites were extracted using a 400 μL methanol/water (4:1, v/v) solution. The mixture was allowed to settle at -20 °C and treated with a high-throughput tissue crusher, Wonbio-96c (Shanghai Wanbo biotechnology co., LTD), at 50 Hz for 6 min, followed by vortexing for 30 s and ultrasound at 40 kHz for 30 min at 5 °C. The samples were placed at -20 °C for 30 min to precipitate the proteins. After centrifugation at 13,000 g at 4 °C for 15 min, the supernatant was carefully transferred to sample vials for LC-MS/MS analysis.

Chromatographic separation of the metabolites was performed on a Thermo UHPLC system equipped with an ACQUITY BEH C18 column (100 mm × 2.1 mm i.d., 1.7 μm; Waters, Milford, USA). The mobile phases consisted of 0.1% formic acid in water (solvent A) and 0.1% formic acid in acetonitrile/isopropanol (1:1, v/v) (solvent B). The solvent gradient changed according to the following conditions to equilibrate the systems: from 0 to 3 min, 95% (A): 5% (B) to 80% (A): 20% (B); from 3 to 9 min, 80% (A): 20% (B) to 5% (A): 95% (B); from 9 to 13 min, 5% (A): 95% (B) to 5% (A): 95% (B); from 13 to 13.1 min, 5% (A): 95% (B) to 95% (A): 5% (B); from 13.1 to 16 min, 95% (A): 5% (B) to 95% (A): 5% (B). The sample injection volume was 2 μL, and the flow rate was set to 0.4 mL/min. The column temperature was maintained at 40°C. During the period of analysis, all these samples were stored at 4 °C.

The mass spectrometric data were collected using a Thermo UHPLC-Q Exactive Mass Spectrometer equipped with an electrospray ionization (ESI) source operating in either positive or negative ion mode. The optimal conditions were set as follows: Aus gas heater temperature, 400 °C; sheath gas flow rate, 40 psi; Aus gas flow rate, 30 psi; ion-spray voltage floating (ISVF), 2800 V in negative mode and 3500 V in positive mode; normalized collision energy, 20-40-60 V rolling for MS/MS. Data acquisition was performed with the Data-Dependent Acquisition (DDA) mode. The detection was carried out over a mass range of 70-1050 m/z.

After UPLC-TOF/MS analyses, the raw data were imported into the Progenesis QI 2.3 (Nonlinear Dynamics, Waters, USA) for peak detection and alignment. The preprocessing results generated a data matrix that consisted of the retention time (RT), mass-to-charge ratio

(m/z) values and peak intensity. Metabolic features detected at least 80 % in any set of samples retained. After filtering, minimum metabolite values were imputed for specific samples in which the metabolite levels fell below the lower limit of quantitation and metabolic features were normalized by sum. The internal standard was used for data QC (reproducibility). Metabolic features for which the relative standard deviation (RSD) of QC > 30% were discarded. Following normalization procedures and imputation, statistical analysis was performed on log-transformed data to identify significant differences in metabolite levels between comparable groups. Mass spectra of these metabolic features were identified using the accurate mass, MS/MS fragments spectra and isotope ratio difference found in reliable biochemical databases, such as the Human Metabolome Database (HMDB) (http://www.hmdb.ca/) and Metlin Database (https://metlin.scripps.edu/). Concretely, the mass tolerance between the measured m/z values and the exact mass of the components of interest was ± 10ppm. For metabolites with MS/MS confirmation, only those with MS/MS fragment scores above 30 were considered as confidently identified. Otherwise, metabolites had only tentative assignments.

A multivariate statistical analysis was performed using the ropls (Version1.6.2, http://bioconductor.org/packages/release/bioc/html/ropls.html) R package from Bioconductor on Majorbio Cloud Platform (https://cloud.majorbio.com). Partial least-squares discriminate analysis (PLS-DA) was used for statistical analysis to determine global metabolic changes between comparable groups. All of the metabolite variables were scaled via Pareto scaling prior to conducting the PLS-DA. The model validity was evaluated from model parameters R2 and Q2, which provide information for the interpretability and predictability, respectively, of the model and avoid the risk of overfitting. Variable importance in the projection(VIP) was calculated in the PLS-DA model. p values were estimated with paired Student's t-test on single-dimensional statistical analysis.

Statistical significance among groups were considered those with a VIP value greater than 1 and a p value less than 0.05 (FDR-corrected). Differential metabolites among the two groups were summarized, and mapped onto their biochemical pathways through metabolic enrichment and pathway analysis based on a database search (KEGG, http://www.genome.jp/kegg/). These metabolites can be classified according to the pathways they involve or the functions they perform. Enrichment analysis is the standard method for analyzing whether appears a group of metabolites appears in a function node or not. The principle is that the annotation analysis of a single metabolite develops into an annotation analysis of a group of metabolites. scipy.stats (Python packages) (https://docs.scipy.org/doc/scipy/) was used to identify statistically significantly enriched pathways using Fisher's exact test.
